# Intraductal Papillary Mucinous Neoplasm of the Pancreas Arising in the Setting of an Intermixed Acinar Cell Cystadenoma of the Pancreas: Report of a Rare Case

**DOI:** 10.1089/crpc.2016.0018

**Published:** 2016-12-01

**Authors:** Benjamin B. Scott, Thea P. Price, Zachary M. Callahan, Justin S. Poling, Harish Lavu

**Affiliations:** ^1^ Department of Surgery, Thomas Jefferson University, Philadelphia, Pennsylvania.; ^2^ Department of Pathology, Johns Hopkins Medical Institutions, Baltimore, Maryland.

**Keywords:** acinar cell cystadenoma, cystic lesions of the pancreas, intraductal papillary mucinous neoplasm, pancreas

## Abstract

**Background:** Synchronous cystic lesions of the pancreas with different pathophysiology in the same patient are a rare occurrence..

**Case Presentation:** We report the incidental finding of a multicystic lesion within the pancreatic head in a morbidly obese woman during workup for bariatric surgery. The lesion contained an intraductal papillary mucinous neoplasm (IPMN) with high-grade dysplasia within an acinar cell cystadenoma (ACA). ACAs are rare tumors first described in 2002.

**Conclusion:** To date, there have been no published reports of synchronous IPMN within an ACA. This case report intends to increase provider awareness of these lesions as well as highlight the importance of surveillance and careful histological examination of heterogeneous cystic lesions of the pancreas.

## Introduction

Synchronous cystic lesions of the pancreas with different pathophysiology in the same patient are a rare occurrence. We report a case of an intraductal papillary mucinous neoplasm (IPMN) with high-grade dysplasia within an acinar cell cystadenoma (ACA). ACA (also known as acinar cystic transformation)^1^ is an extremely rare process, first described in the literature in 2002,^2^ and to our knowledge there have been no published reports of a synchronous IPMN within an ACA. The aim of this case report is to describe an extremely rare and remarkable case, to increase provider awareness of these lesions, and to highlight the importance of surveillance and careful histopathological examination of heterogeneous cystic lesions of the pancreas.

## Case Report

**FIG. 1.**
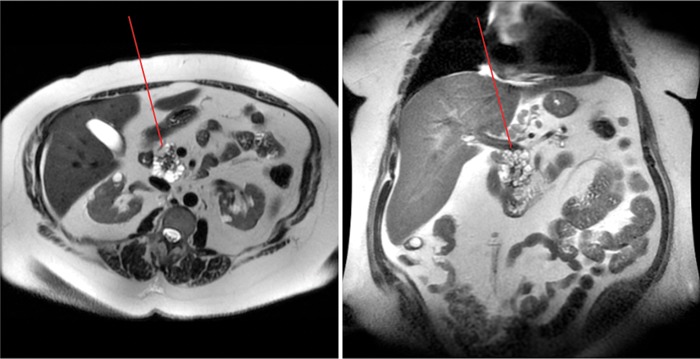
MRI of the abdomen of the 53-year-old woman 1 month before surgery reveals a cystic mass in the head of the pancreas (arrow) with distal pancreatic atrophy. Periportal and peripancreatic lymphadenopathy is appreciated.

A 53-year-old woman presented with progression of a known cystic lesion within the head and uncinate process of the pancreas. After a diagnosis of type 2 diabetes in 2006, an MRI of the abdomen revealed a 4.4 × 4.1 × 4.7 cm unilocular nonenhancing cystic mass. In 2015, the patient underwent a second MRI of the abdomen as part of a workup for weight-reduction surgery, which showed a centrally enhancing, multilobulated, and septate appearing cystic mass measuring 5.0 × 4.4 × 5.8 cm. Additional findings included several nonenhancing subcentimeter cystic lesions in the body of the pancreas, atrophy of the tail, and periportal adenopathy with nodes measuring up to 3.5 × 1.9 × 4.8 cm (Fig. 1). Endoscopic ultrasonography confirmed the presence of mural nodularity within the cystic tumor. Preoperative laboratories were notable for a slightly elevated cancer antigen 19.9 of 39 U/mL (normal range: <35 U/mL). Given the size of the cyst, mural nodularity, and associated periportal adenopathy, there was a suspicion of potential malignant degeneration of the tumor. The patient was advised to undergo surgical resection and this was successfully accomplished with a pylorus-preserving pancreaticoduodenectomy with standard reconstruction as previously described.^3^ The patient tolerated the procedure well, had an uneventful hospitalization, and was discharged to home on postoperative day 7.

Postoperative histological sections showed cystic dilation of the main pancreatic duct and side branch ducts. The majority of these ducts were lined by papillary mucinous epithelium consistent with IPMN and showed focal areas of high-grade dysplasia, characterized by cribriform architecture, complex papillary fronds, nuclear stratification, nuclear hyperchromasia, and loss of nuclear polarity. There was also a moderate degree of periductal parenchymal fibrosis and atrophy, indicative of chronic pancreatitis. All ducts retained a lobular configuration, precluding the diagnosis of invasive carcinoma.

**FIG. 2.**
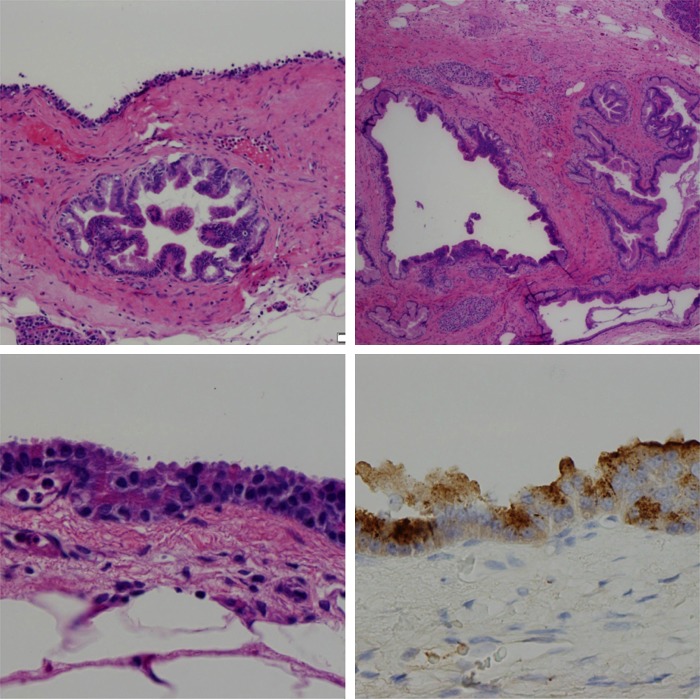
Top left: A focus of acinar cystadenoma superiorly abutting a focus of IPMN. Hematoxylin and eosin stain (100 μm); top right: IPMN with focal high-grade dysplasia. Papillae growing into the ducts—classic of IPMN histology (200 μm); bottom left: granular red cytoplasm showing acinar differentiation and a single prominent nucleus (a feature of acinar cells) (20 μm); bottom right: chymotrypsin stain clearly showing brown appearing zymogen granules from acinar cells (20 μm). IPMN, intraductal papillary mucinous neoplasm.

Admixed within the ducts lined by mucinous epithelium were cysts lined by cuboidal epithelium with prominent eosinophilic cytoplasmic zymogen granules and prominent nucleoli, consistent with an ACA. These cysts showed strong immunohistochemical labeling for trypsin, further supporting an acinar origin; the cysts lined by mucinous epithelium, however, were negative for trypsin. There was no evidence of dysplasia within the acinar epithelium (Fig. 2).

The striking morphological differences between the two epithelial cyst linings supported the diagnosis of synchronous lesions, with distinct components of both an IPMN and ACA. Both processes seemed to contribute to pancreatic duct dilation.

## Discussion

Cystic lesions of the pancreas can be separated into true cysts, pseudocysts, and cystic neoplasms. ACAs were first described in 2002 as a rare cystic neoplasm distinguished histologically by the presence of a single layer of cuboidal or low columnar acinar cell epithelium.^1^ ACA can be further differentiated from other pancreatic neoplasms with immunohistochemistry; ACA positively labels with antibodies against trypsin and chymotrypsin,^4^ as was the case in our patient. Recent studies have shown slightly higher incidence in female gender (61%) and a median age of 49.5.^4^ This age is ∼10 years before the median age of incidence for both ACA and acinar cell carcinoma. The earlier incidence of ACA is similar to the age of incidence of premalignant lesions of the pancreas such as IPMN.^4^ The percentage of IPMNs found to harbor malignancy varies with location; malignant cells are found in 43% of main duct-IPMNs but only in 18% of branch duct-IPMNs.^5^ Owing to poor understanding of the molecular biology of ACA, there is debate over ACA being considered a neoplastic or a non-neoplastic process. A recent study of ACA involving mitochondrial DNA and clustering analyses failed to demonstrate a common clonal origin, lending credence to the argument that ACA should be considered non-neoplastic.^6^ However, a recent report of 10 cases of ACA found 2 cases in which intramural nodules were identified; these nodules were later found to have chromosomal imbalances, indicative of a neoplastic process.^7^ Currently, because of its potential for malignancy, the presence of ACA warrants surgical excision.^8^ Awareness of ACA should be present among clinical providers when evaluating asymptomatic cystic lesions of the pancreas. The possibility of synchronous pancreatic cystic lesions also adds to sampling error during preoperative workup, as a “benign” cystic biopsy could still harbor premalignant lesions within the remaining mass.^9,10^ Because pancreatic carcinoma continues to carry a dire prognosis despite surgical and adjuvant advances, the shift in recent years toward resection of premalignant lesions has become paramount; we present this new finding to increase awareness of synchronous pancreatic lesions.

## Abbreviations Used

ACA: acinar cell cystadenoma
IPMN: intraductal papillary mucinous neoplasm
MRI: magnetic resonance imaging
